# Saddle embolism is associated with the major adverse events in patients with nonhigh-risk pulmonary embolism

**DOI:** 10.1186/cc11026

**Published:** 2012-03-20

**Authors:** H Kim, W Kim

**Affiliations:** 1 Asan Medical Center, Seoul, South Korea

## Introduction

In some patients with acute pulmonary embolism (PE), thrombi may lodge at the levels of bifurcation of the pulmonary trunk and extend into both main pulmonary arteries, forming so-called saddle embolism (SE). The aim of this study was to assess the incidence of SE and whether it is associated with an increased risk of complicated clinical course in patients with nonhigh-risk PE.

## Methods

Between January 2006 and June 2010, 297 consecutive patients with nonhigh-risk PE that was confirmed with contrast-enhanced spiral computed tomography (CT) in the emergency department were studied. One experienced radiologist evaluated the presence of SE. The clinical information, echocardiographic and CT parameters were reviewed. Patients were divided into SE and non-SE. Multivariate logistic regression was applied to determine factors associated with occurrence of major adverse events (MAE).

## Results

Twenty-seven out of 297 patients (9.1%) were found to have a SE. Overall mortality at 1 month was 12.5% with no difference between the groups (11.9% vs. 18.5%, *P *= 0.32), although SE patients were more likely to receive thrombolytic therapy (8.1% vs. 29.6%, *P *< 0.01). SE patients had s significantly higher rate of MAE (59.3% vs. 25.6%, *P *< 0.01). Presence of SE and the ratio of right ventricular to left ventricular diameter were associated with an odds ratio of MAE within 1 month of 2.48 (95% CI: 1.10 to 6.04, *P *= 0.03) and 3.34 (95% CI: 1.46 to 7.46, *P *< 0.01).

## Conclusion

SE by CT angiography was associated with PE-related shock, intubation, mortality, thrombolysis, and thrombectomy within 1 month in patients with nonhigh-risk PE and may be a useful method for simple risk stratification.

